# Fault Diagnosis for Rail Vehicle Axle-Box Bearings Based on Energy Feature Reconstruction and Composite Multiscale Permutation Entropy

**DOI:** 10.3390/e21090865

**Published:** 2019-09-05

**Authors:** Xiaochao Wang, Zhenggang Lu, Juyao Wei, Yuan Zhang

**Affiliations:** Institute of Rail Transit, Tongji University, Shanghai 201804, China

**Keywords:** axle-box bearing of rail vehicle, wavelet packet transform, energy feature reconstruction, composite multiscale permutation entropy, MG-SVM, fault diagnosis

## Abstract

The fault response signals of an axle-box bearing of a rail vehicle have strongly non-linear and non-stationary characteristics, which can reflect the operating state of the running gears. This paper proposes a novel method for bearing fault diagnosis based on frequency-domain energy feature reconstruction (EFR) and composite multiscale permutation entropy (CMPE). First, a wavelet packet transform (WPT) is applied to decompose the vibration signals into multiple frequency bands. Then, considering that the bearing-localized defects cause the axle-box bearing system to resonate at a high frequency, which will lead to uneven energy distribution of the signal in the frequency domain, the energy factors of each frequency band are calculated by an energy feature extraction algorithm, from which the frequency band with maximum energy factor (which contains abundant fault information) is reconstructed to the time-domain signal. Next, the complexity of the reconstructed signals is calculated by CMPE as fault feature vectors. Finally, the feature vectors are input into a medium Gaussian support vector machine (MG-SVM) for bearing condition classification. The proposed method is validated by a public bearing data set and a wheelset-bearing system test bench data set. The experimental results indicate that the proposed method can effectively extract bearing fault features and provides a new solution for condition monitoring and fault diagnosis of rail vehicle axle-box bearings.

## 1. Introduction

As an important component affecting the operational safety of rail vehicles, the axle-box bearing of the running gear bears various dynamic impacts, such as vehicle body load and starting, traction, and braking forces during operation. At the same time, the surfaces of many components inside the bearing are in contact with each other, causing the bearing to generate localized defects, such as inner race, roller, outer race, and cage faults. As the axle-box bearing is affected by the wheel-rail high frequency impact and the alternating load of the primary suspension, the vibration signals show strongly non-linear and non-stationary characteristics, and the response signal of an early fault of a bearing is very weak, relative to the strong background noise. Extracting the features of bearing faults in the non-linear, multi-component amplitude and frequency modulated signals, and accurately identifying the bearing conditions has always been a difficult point in bearing fault diagnosis [1,2,3].

Bearing fault diagnosis consists of two aspects: fault feature extraction and fault type recognition. Scholars have carried out many studies on these two aspects and proposed different theoretical algorithms. Time-frequency analysis methods can be used effectively to decompose and describe response signals of bearing early faults, including wavelet transform (WT), empirical mode decomposition (EMD) [4], local mean decomposition (LMD) [5], empirical wavelet transform (EWT) [6], and variational mode decomposition (VMD) [7] methods. WT is an effective time-frequency analysis method which has a good noise reduction effect [8], but it only decomposes the low-frequency band of the signal; the high-frequency band is not processed, so, the frequency resolution in the high-frequency band is low. Wavelet packet transform (WPT) methods improve on the wavelet transform, which can decompose the high- and low-frequency bands of the signal into multiple layers and provide a high-resolution analysis method for the signal. At the same time, according to the characteristics of the analyzed signal, the corresponding frequency bands can be adaptively selected to match the original signal spectrum [9]. EMD is an effective time-frequency analysis approach, which can decompose complicated multi-component signals into a set of intrinsic mode functions (IMFs). In order to overcome the drawbacks of EMD, such as the boundary effect, mode mixing, and under- and over-shoot problems, improved methods, including ensemble EMD (EEMD) [10], complementary EEMD (CEEMD) [11], and complete EEMD with adaptive noise (CEEMDAN) [12] have been proposed. In [13,14,15], the CEEMDAN and VMD methods have been applied for feature extraction and denoising of underwater acoustic signals.

EMD and its derivative methods, LMD, and VMD methods can decompose complicated signals self-adaptively; however, the signal frequency bands cannot be accurately divided, and the IMF decomposition results are related to the original signal characteristics, which cannot form a uniform frequency distribution. WPT can decompose a signal with multiple scales and high resolution based on the frequency distribution with more uniform frequency feature extraction results, which is beneficial to the unified feature extraction of different frequency bands of fault-impact signals and facilitates intelligent classification of multiple sets of signals. Fan [16] proposed a wavelet-based statistical signal detection approach for monitoring and diagnosis of bearing compound faults at an early stage. Bin [17] combined WPT and EMD to extract fault feature frequencies for early fault feature extraction in rotating machinery. Bastami [18] used WPT to extract vibration signal features and applied an artificial neural network to estimate the remaining life of rolling element bearings. Wang [19] proposed a novel sparse wavelet reconstruction residual feature for rolling element bearing diagnosis, based on WPT and sparse representation theory. Wan [20] used the binary wavelet packet transform, instead of the finite impulse response filter bank, as the frequency band segmentation method for optimizing the fast spectrum kurtosis algorithm. As localized bearing defects cause the energy of vibration signals to change in the frequency domain, the features of the frequency-domain energy can describe the vibration signal [21]. Huang [22] decomposed bearing vibration signals into wavelet signals of different frequency bands, where different frequency band signals were respectively reconstructed to extract energy features, which formed the feature vectors for input into classification models. Ma [23] extracted the energy distribution coefficients and energy entropy of the third-layer wavelet packet decomposition coefficients as the characteristic parameters of the subsequent classification models. In conclusion, many studies have shown that WPT can self-adaptively decompose a signal into different frequency bands and that the energy factors of each frequency band can reflect the intrinsic features of the signals. This paper is inspired by this conclusion and, as such, proposes a primary feature extraction method based on WPT and energy feature reconstruction.

In recent years, various entropy-based complexity measurement methods deriving from information theory have been proposed for feature extraction of non-linear vibration signals [24,25], such as approximate entropy (ApEn) [26], sample entropy (SampEn) [27], multiscale entropy (MSE) [28], and fuzzy entropy [29]. Considering that a combination of resonances can change the pattern of shaft vibration responses, Nicoletti [30] used the ApEn algorithm to highlight the presence of such resonances and detected the incipient cracks. Li [31] proposed a novel signal processing method by combining the improved Vold–Kalman filter and multiscale sample entropy for planetary gearboxes under non-stationary working conditions. The simulation and experimental results showed that the presented method had superior performance in identifying fault types of planetary gearboxes. Hsieh [32] combined empirical mode decomposition and MSE to extract the fault defects of a high-speed Spindle. Zheng [33] proposed a Sigmoid-based refined composite multiscale fuzzy entropy method and used it for dynamical complexity analysis of mechanical vibration. The analysis and results showed that the proposed method had a better distinguishing capacity and robustness, as well as being capable of reflecting more complex information of the time-series. Permutation entropy (PE) was proposed by Bandit and Pompe [34], which has the superiority of robustness, simplicity, computational efficiency, and invariance to non-linear monotonous transformations. For rotary machinery vibration signal fault feature extraction, Zheng [35] combined PE and VMD, Xiao [36] combined PE and smooth local subspace projection, and Tian combined [37] PE and manifold-based dynamic time warping; all of which could diagnose faults accurately. However, the PE method only has a single scale, which makes it insufficient when dealing with the dynamic changes of vibration signals. In order to overcome the drawbacks of the PE method, Aziz and Arif [38] proposed a new method, named multiscale permutation entropy (MPE), using a combination of MSE and PE. MPE can extract features from time-series signals at different scales and has better robustness, strong anti-noise capability, and can effectively reflect the characteristics of the non-linear dynamics and random vibrations of a rolling bearing. Zhang [25] used MPE to calculate the complexity of a reconstructed feature space signal. Zheng [39] proposed an improved MPE method, called generalized composite multiscale permutation entropy, to solve the drawback of the coarse graining process in MPE. To improve the trend and stability of the MPE method, composite multiscale permutation entropy (CMPE) was put forward by improving the coarse-grained procedure and obtaining several PE values to describe in one same scale [40,41]. Si [42] applied CMPE for accurate cutting state recognition of a shearer and Yin [43] combined CMPE and WPT for arc fault detection; they all achieved expected results, which proved the effectiveness of feature extraction by CMPE.

In this paper, a novel method for the fault diagnosis of rail vehicle axle-box bearings is proposed, based on frequency-domain energy feature reconstruction (EFR) and CMPE. According to mechanical vibration theory, a localized bearing defect can cause the axle-box bearing system to resonate at a high frequency, which can lead to an uneven energy distribution of the vibration signal in the frequency domain. Firstly, the bearing vibration signals are decomposed by a three-layer WPT to divide the original signals into eight parts, from low frequency to high frequency. Secondly, the energy factors of each frequency band are calculated by the energy feature extraction algorithm, and the frequency band containing abundant fault information with maximum energy factor is reconstructed to the time-domain signal. Thirdly, the complexity of the reconstructed signal is calculated by CMPE over multiple time scales to obtain a series of PE, which can describe the features of original vibration signal. Finally, as the fault feature matrixes, the CMPEs are input into a medium Gaussian support vector machine (MG-SVM) for bearing condition classification.

The rest of this paper is organized as follows: Section 2 reviews the WPT and energy extraction algorithm, following which the energy feature reconstruction criterion is proposed. The PE, MPE, and CMPE methods are introduced in Section 3. The proposed method flow is described in Section 4, which is validated and analyzed by multiple experiment data sets in Section 5. The discussion and conclusion are given in Section 6 and Section 7, respectively. The significations of the acronyms for the different algorithms in this paper are listed in Table A1, in Appendix A.

## 2. WPT-Based Energy Feature Reconstruction

### 2.1. WPT and Energy Feature Extraction Algorithm

The continuous wavelet transform of a finite-energy signal ($x(t)\in L^{2}(R)$) is [17]

(1) $$W_{x}(a,b)=\frac{1}{\sqrt{\left|a\right|}}\int_{-\infty }^{+\infty }x(t)\varphi^{*}(\frac{t-b}{a})dt,$$

where $\varphi^{*}(t)$ is the conjugated wavelet of the mother wavelet φ(t), a is the scale parameter, b is the translation parameter, the factor $1/\sqrt{\left|a\right|}$ is used for energy preservation, and $W_{x}(a,b)$ is the continuous wavelet transform of x(t).

The scale parameter can adjust the shape of φ(t) and the translation parameter can adjust the displacement of φ(t), which makes wavelet analysis an effective decomposition method for a non-linear and non-stationary signal in both the time and frequency domains.

Although the wavelet analysis is an effective method for time-frequency analysis, it only decomposes the low-frequency part—the high-frequency part is not processed—which makes the frequency resolution of high-frequency part poor. However, WPT, which was derived from WT, can decompose both the low- and high-frequency parts. Thus, the WPT is a full-scale time-frequency analysis method.

**Figure 1:**
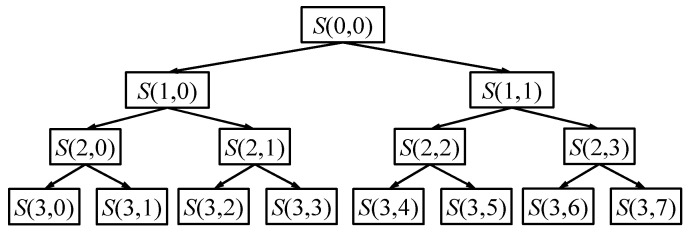
Schematic of a three-layer wavelet packet decomposition.

The two-scale function of WPT, including a scaling function (ϕ(t)) and wavelet function (φ(t)) can be expressed as

(2) $$\phi (t)=\sqrt{2}\sum_{k}h(k)\phi (2t-k)$$

(3) $$\varphi (t)=\sqrt{2}\sum_{k}g(k)\phi (2t-k),$$

where h(k) is the low-pass filter and g(k) is the high-pass filter. Based on the wavelet filters, the signal is decomposed into a form of a binary tree. The WPT coefficients can be expressed as

(4) $$d_{j+1,\;2n}=\sum_{m}h(m-2k)d_{j,\;n}$$

(5) $$d_{j+1,\;2n+1}=\sum_{m}g(m-2k)d_{j,\;n},$$

where j is the decomposition layer, n is the node number in layer j, and m is the number of wavelet coefficients. Figure 1 is a binary tree of a three-layer WPT, where S(0,0) represents the original signal, and the signal is decomposed into eight sub-bands.

WPT decomposes a signal into different frequency bands. When a bearing fault occurs, the localized defect impact can cause resonance at a different natural frequency from the axle-box bearing system, resulting in an uneven energy distribution in the frequency domain of the vibration signal. Therefore, energy distribution regularity in the frequency domain can be viewed as an important characteristic of a vibration signal. For an orthogonal wavelet packet space, the energy factor (E(j,n)) of the frequency domain in the signal WPT space is defined as [21]

(6) $$E(j,n)={\sum \left[d(j,n)\right]}^{2},$$

where d(j,n) is the WPT coefficient, which can be calculated by Equations (4) and (5).

If the original signal is subjected to a J-layer wavelet packet full-scale decomposition, the energy characteristic matrix (C(J,s)) of each frequency band under the J-layer WPT can be calculated as

(7) $$C(J,s)=[E(J,2^{0}),\;E(J,2^{1}),\;\cdots ,\;E(J,2^{J}-1)].$$

When an axle-box bearing has a fault, the localized defects will cause the axle-box bearing system to resonate at a high frequency, which will lead to uneven energy distribution of the signal in the frequency domain and frequency bands with higher energy factors contain more fault feature information. The wavelet packet reconstruction is performed on the frequency band with maximum energy factor and, then, a new time-domain vibration signal with more obvious fault features is obtained.

### 2.2. Experimental Verification

**Figure 2:**
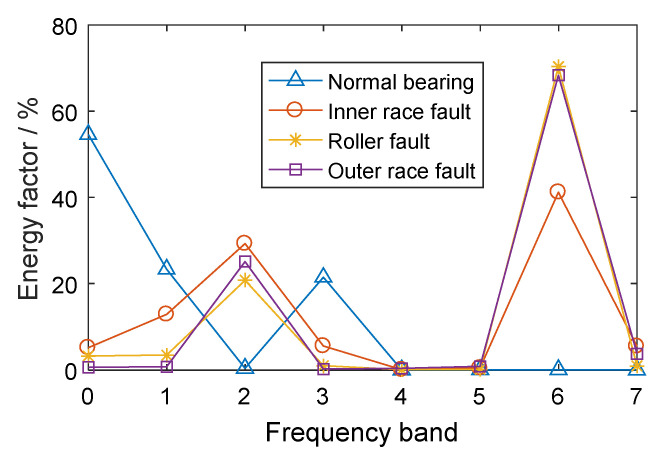
The energy factors of frequency bands for rolling bearings in different conditions.

The CWRU-BDC (Case Western Reserve University Bearing Data Center) data set is taken as an analysis example (this data set will be introduced in more detail in Section 5.1). Based on the experimental vibration signals in different conditions, including a normal bearing, a bearing with an inner race fault, a bearing with a roller fault, and a bearing with an outer race fault, three-layer WPT was performed on the vibration signals. Each signal was decomposed into eight parts in the frequency domain and the energy factors were calculated according to Equation (6). The energy distributions of the different bearings are shown in Figure 2.

It can be seen, from Figure 2, that there were differences in the energy distributions of bearings in different conditions. For the normal bearing, the signal energy was mainly concentrated in the low frequency band (especially in the 0th frequency bands) and the energy factor in the high frequency band was relatively small. The energy of the low frequency band for the normal bearing mainly came from the even vibration noise generated by the rotation of the bearing. The frequency band energy distributions of the bearings with different kinds of faults were similar, mainly concentrated in the 2nd and 6th frequency bands, especially the 6th frequency band of the high frequency part, which was caused by the fact that localized defect impact can cause resonance at high natural frequencies of the axle-box bearing system. Therefore, for the fault bearings, the 6th frequency band, with high energy factor, contained abundant fault feature information. The normal bearing energy in the 6th frequency band was small, such that the normal bearing and the fault bearings had significant feature differences in this frequency band.

**Figure 3:**
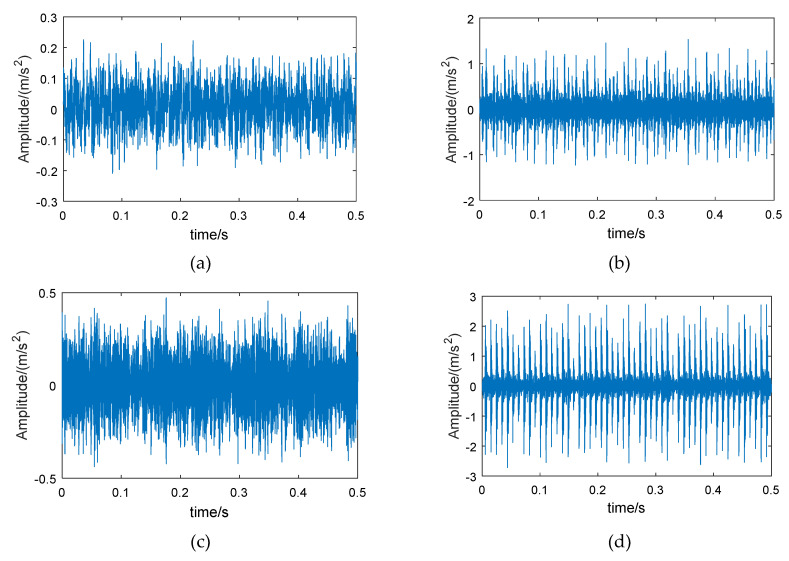
Vibration signals of different bearings: (**a**) normal bearing, (**b**) bearing with inner race fault, (**c**) bearing with roller fault, and (**d**) bearing with outer race fault.

**Figure 4:**
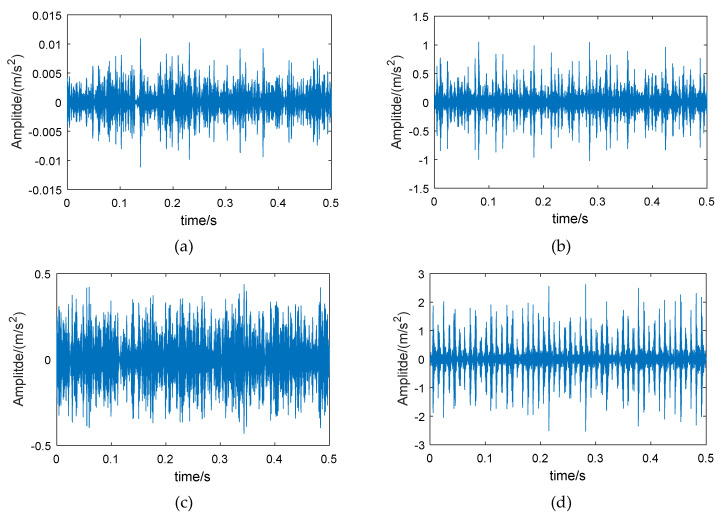
The 6th frequency band reconstructed signals of different bearings, based on energy feature reconstruction: (**a**) normal bearing, (**b**) bearing with inner race fault, (**c**) bearing with roller fault, and (**d**) bearing with outer race fault.

Therefore, wavelet packet reconstruction can be performed on the high-energy frequency band of the fault bearing, from which time-domain vibration signals with more bearing feature information are obtained. Figure 3 shows the original vibration signals of the bearing in different conditions, and Figure 4 shows the reconstructed signals of the 6th frequency band.

Comparing Figure 3 and Figure 4, it can be seen that the amplitude of the reconstructed signal for the normal bearing was significantly reduced, but the reconstruction signals for the fault bearings showed less loss in amplitude, such that the difference between the normal bearing and the fault bearings was more obvious. On the other hand, the low-amplitude noise of the reconstructed fault signals was reduced, which is due to the fact that the low frequency bands were excluded during signal reconstruction.

**Figure 5:**
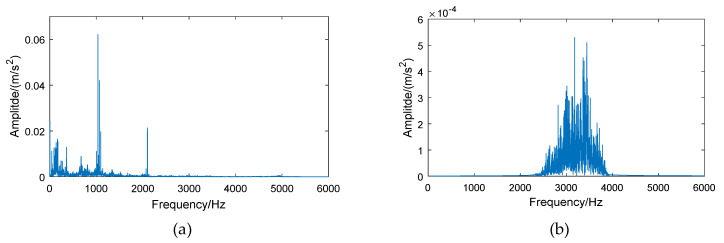
Spectrum of vibration signals for a normal bearing: (**a**) the original signal, (**b**) the reconstructed signal of the 6th frequency band.

**Figure 6:**
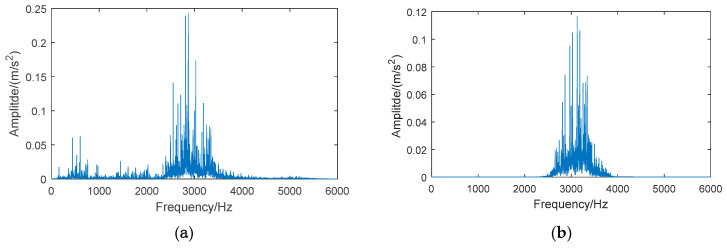
Spectrum of vibration signals for a bearing with inner race fault: (**a**) the original signal, (**b**) the reconstructed signal of the 6th frequency band.

Figure 5 and Figure 6 show the signal spectrum of the normal bearing and inner race fault bearing before and after energy feature reconstruction. It can be seen, from Figure 5, that the energy of normal bearing is mainly distributed at low frequency segment (below 2 kHz) and the reconstructed signal of the 6th frequency band is located at 2–4 kHz, where the energy of original signal is small, such that the frequency amplitude of reconstructed signal is greatly reduced. In Figure 6, it can be seen that the signal frequency of bearing with inner race fault is mainly distributed in 2–4 kHz, which contains abundant bearing fault information. After the signal reconstruction of the 6th frequency band by the wavelet packet, the main energy of original signal is saved. At the same time, the noise at low frequency is suppressed and the loss of frequency amplitude for reconstructed signal is small. The above analysis further demonstrates that the method of energy feature reconstruction can effectively extract the bearing fault information, and suppress the signal energy of the normal bearing, which can increase the signal differences between the normal bearings and the fault bearings.

## 3. Composite Multiscale Permutation Entropy

### 3.1. Permutation Entropy

Permutation entropy (PE) was proposed for measuring the complexity and detecting the chaotic dynamic change of a time-series by comparing adjacent values without considering the size of the values, which makes it suitable for analyzing non-stationary time-series detected from complex dynamic systems.

Considering a given time-series {x(i), i=1,2,…,N}, it can be reconstructed in phase space by [34]:

(8) $$\left\{ \begin{array}{l} X(1)=\left\{x(1),x(1+\lambda ),\ldots ,x(1+(m-1)\lambda )\right\}\\ \vdots \\ X(i)=\left\{x(i),x(i+\lambda ),\ldots ,x(i+(m-1)\lambda )\right\}\\ \vdots \\ X(N-(m-1)\lambda )=\left\{x(N-(m-1)\lambda ),x(N-(m-2)\lambda ),\ldots ,x(N)\right\} \end{array}\right.,$$

where m is the embedding dimension and λ is the time delay. X(i) is the reconstructed series in phase space. The elements in X(i) are sorted by increasing order:

(9) $$X_{S}(i)=\{x(i+(j_{1}-1)\lambda )\leq x(i+(j_{2}-1)\lambda )\leq \ldots \leq x(i+(j_{m}-1)\lambda )\},$$

where $X_{S}(i)$ is the sorted series. j is the position order of x(i) in X(i) and 1≤j≤m.

For an arbitrary series X(i), there are m! permutation possibilities, and the relative frequency of each permutation π can be obtained by

(10) $$p(\pi )=\frac{Number\{i|i\leq N-m,\;X_{i}^{m}=\pi \}}{N-(m-1)\lambda },$$

where $X_{i}^{m}$ is one of $X_{S}$ and the elements sorting order of $X_{i}^{m}$ is same with permutation π when embedding dimension is m.

Based on Shannon entropy, the PE of dimension m can be defined as

(11) $$H_{PE}(m)=-\sum_{\pi =1}^{\pi =m!}p(\pi )\ln(p(\pi )).$$

The maximum of $H_{PE}(m)$ can reach ln(m!), which is related with the dimension m. In general, $H_{PE}(m)$ can be normalized as

(12) $$H_{PE}=H_{PE}(m)/\ln(m!).$$

Thus, PE ranging from 0 to 1 can be calculated by the above procedure. If $H_{PE}$ is small, it means that the series is regular; if $H_{PE}$ is large, it means the series is random.

### 3.2. MPE and CMPE

MPE and CMPE are improved methods derived from PE. In order to solve the shortcomings of PE’s single-scale analysis of signal, a coarse-grained procedure was put forward to generate multiple related series based on a signal series. As one PE value can be calculated through one series, MPE and CMPE can use a series of PE values to describe one series, which can yield more information for a complex time-series. The difference between MPE and CMPE is in the coarse-grained procedure.

(1) Coarse-grained procedure of MPE:

Considering a given time-series {x(i), i=1,2,…,N} of length *N*, each new time-series $y^{\left(\tau \right)}$ can be obtained by calculating the average of successive data within non-overlapping windows at a scale factor of τ:

(13) $$y_{j}^{\left(\tau \right)}=\frac{1}{\tau }\sum_{i=(j-1)\tau +1}^{j\tau }x_{i}\;1\leq j\leq \left\lfloor \frac{N}{\tau }\right\rfloor ,$$

where τ is the scale factor and ⌊N/τ⌋ is the length of each of the coarse-grained series $y^{\left(\tau \right)}$.

Then, each coarse-grained series $y^{\left(\tau \right)}$ with different scale factor τ can be calculated and obtain a PE value. Multiple PE values constitute the MPE:

(14) $$MPE(X,\tau ,m,\lambda )=PE(y^{\left(\tau \right)},m,\lambda ),$$

where m is the embedding dimension and λ is the time delay.

Although MPE can describe the complexity of a time-series through multiple PE values, which is better than one single PE, the conventional MPE method still has two drawbacks: firstly, the coarse-grained procedure of MPE is not symmetric. Secondly, the MPE results have unstable trends over for long temporal scales.

(2) Coarse-grained procedure of CMPE:

CMPE improves the coarse-grained procedure by using multiple coarse-grained time-series at one scale factor. By improving Equation (13), CMPE obtains τ new coarse-grained series at a scale factor of τ, where the k*^th^* coarse-grained series can be obtained by:

(15) $$y_{k,j}^{\left(\tau \right)}=\frac{1}{\tau }\sum_{i=(j-1)\tau +k}^{j\tau +k-1}x_{i}\;1\leq j\leq \left\lfloor \frac{N}{\tau }\right\rfloor ,\;1\leq k\leq \tau .$$

Then, the average of multiple PE of τ new coarse-grained series is calculated to obtain the CMPE value at a scale factor τ:

(16) $$CMPE(X,\tau ,m,\lambda )=\frac{1}{\tau }\sum_{k=1}^{\tau }PE(y_{k}^{\left(\tau \right)},m,\lambda ).$$

**Figure 7:**
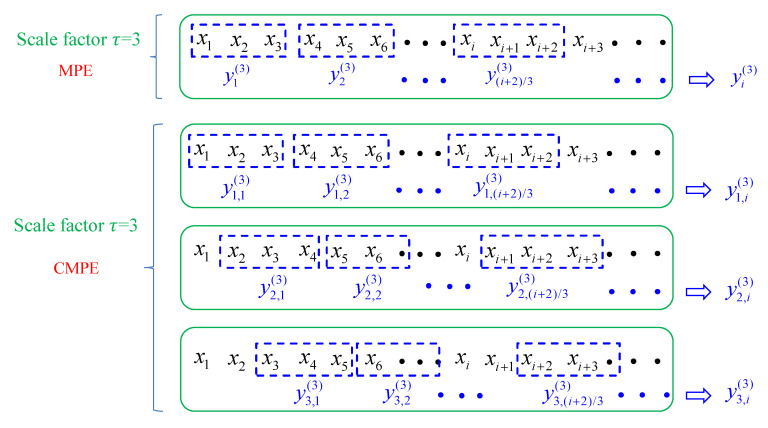
The coarse-grained procedures of composite multiscale permutation entropy (CMPE) and multiscale permutation entropy (MPE) with scale factor 3.

With a scale factor of 3, the coarse-grained procedures of MPE and CMPE are shown in Figure 7. MPE can decompose one time-series at a scale factor of 3, while the CMPE can decompose three time-series at a scale factor of 3.

### 3.3. Simulation Contrast between MPE and CMPE

**Figure 8:**
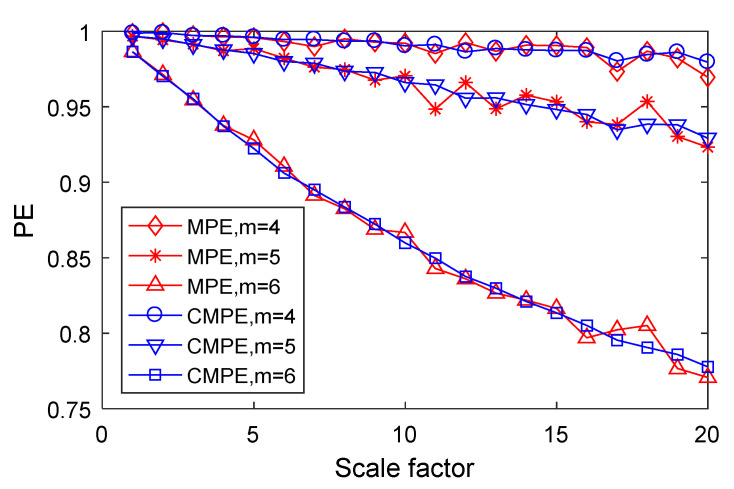
Contrast between CMPE and MPE over different embedding dimension and scale factors.

In order to contrast and compare CMPE and MPE, 4096 Gaussian white noise data points were taken as the extraction object, the scale factor was set to 20, the embedding dimension to 4–6, and the delay time to 1. Then, using CMPE and MPE, the PE values of the Gaussian white noise data were found. The result is shown in Figure 8.

In Figure 8, it can be seen that, as the scale factor increased, the value of PE decreased. Furthermore, the larger the embedding dimension was, the faster the PE decreased. This is because the data length reduces as the scale factor increases and, so, the time-series becomes more stable and the PE decreases. The smaller the embedding dimension is, the shorter the length of the phase space reconstruction signal. The time-series becomes more complex, which leads to an increase in PE. However, comparing the extraction results of CMPE and MPE, it can be seen that the CMPE calculation results were obviously more stable.

## 4. The Proposed Fault Diagnosis Method

Based on the signal feature analysis of fault bearings and non-linear time-frequency analysis algorithms, this paper combines WPT, EFR, CMPE, and the classification method of MG-SVM (WPT-EFR-CMPE + MG-SVM) to propose the following fault diagnosis process for rail vehicle axle-box bearings:

(1) Perform three-layer dmey kernel (discrete Meyer wavelet) WPT on the vibration signals of the training data set and decompose the signals into eight frequency bands.

(2) Calculate the energy factor of each frequency band using the wavelet packet coefficients. The position of the frequency band with the highest energy factor of the fault signal will be chosen, and all frequency bands at this position are constructed to time-domain signals.

(3) For the reconstructed time-domain signals, calculate their CMPE values and set the scale factor to 15, the embedding dimension to 6, and the delay time to 1. Each signal is transformed into a feature vector containing 30 factors.

(4) Input the feature vectors into the MG-SVM to establish a bearing condition classifier through supervised learning.

(5) For the test data set, extract their features using steps 1 to 3. Then, input the features into the classifier for bearing condition classification and fault size classification.

**Figure 9:**
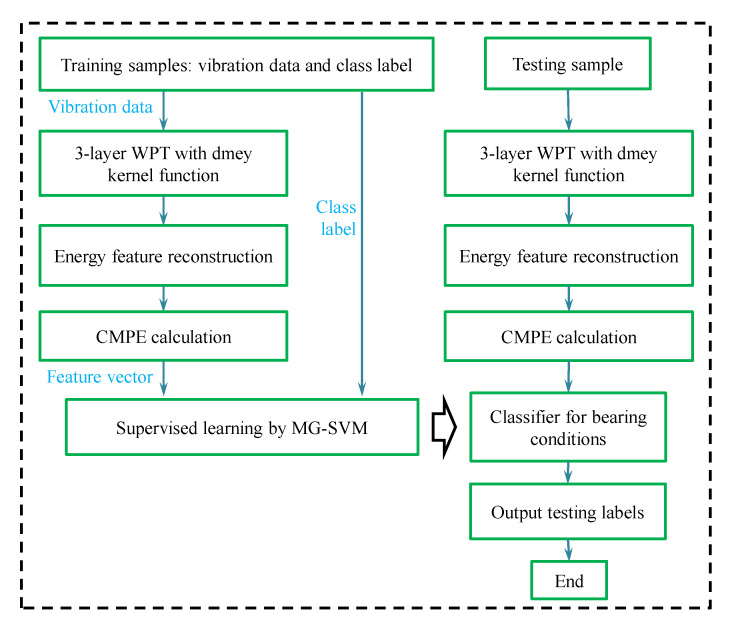
The flowchart of bearing fault diagnosis based on a wavelet packet transform, energy feature reconstruction, composite multiscale permutation entropy and classification method of medium Gaussian support vector machine (WPT-EFR-CMPE + MG-SVM).

Figure 9 shows the flowchart of the proposed method.

## 5. Experimental Results and Analysis

### 5.1. Experimental Validation 1

**Figure 10:**
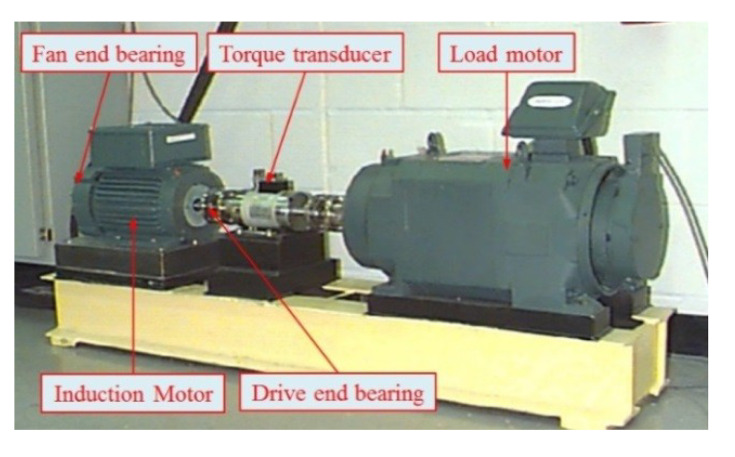
The test bench (CWRU-BDC—Case Western Reserve University Bearing Data Center) for fault diagnosis of a rolling bearing.

**Figure 11:**
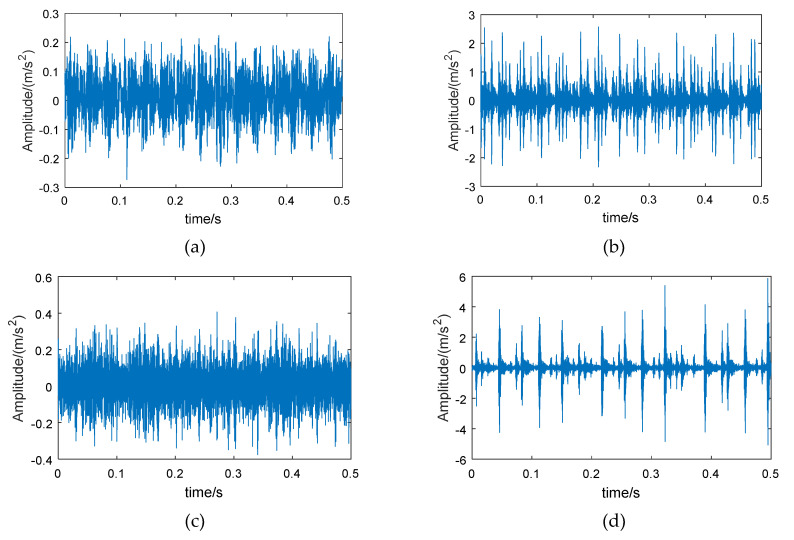
Vibration signals of different bearings (from CWRU-BDC): (**a**) normal bearing, (**b**) bearing with inner race fault, (**c**) bearing with roller fault, and (**d**) bearing with outer race fault.

The method proposed in this paper was verified using the bearing test bench data set from CWRU-BDC (Case Western Reserve University Bearing Data Center) [44]. The test bench is shown in Figure 10. The test bearing was a SKF6205-2RS deep groove ball bearing, which included four conditions: normal bearing (Normal), inner race fault (IRF), roller fault (BF), and outer race fault (ORF). The vibration signal sampling frequency was 12 kHz. In order to increase the fault diagnosis difficulty, the rotation speeds of the bearing corresponding to the chosen data were 730, 1750, 1772, and 1797 rpm. Figure 11 shows an example of the time-domain waveforms of vibration signals corresponding to different bearing conditions. The normal bearing has no obvious fault impact (Figure 11a), and the amplitude fluctuates within a small range; the time-domain waveform of the bearing with roller fault is similar to normal bearing (Figure 11b), but the vibration amplitude is slightly larger, indicating that the impact vibration caused by the roller was small and easily covered by noise; the inner race fault and the outer race fault bearings have obvious periodic impact characteristics (Figure 11c,d), and the waveform amplitude is large.

The training and testing data set samples were collated, where each sample consisted of 2048 points containing information for approximately five cycles of the bearing. For each fault type, the fault point diameter was selected from 0.1778, 0.3556, or 0.5334 mm. For each fault diameter, 12 training samples and 48 testing samples were selected. The sample labels were divided into two groups. Group I was in accordance with the bearing fault types and contained four categories, and Group II contained 10 categories in accordance with the fault diameters of bearings in different conditions. Detailed information of the data set is shown in Table 1.

**Figure 12:**
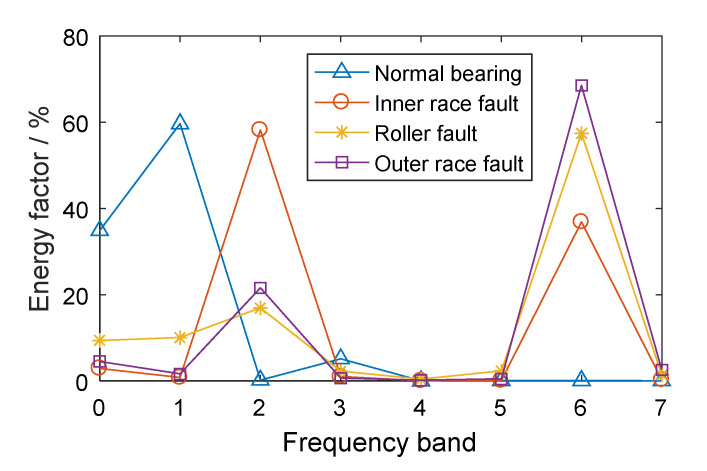
The energy factors of frequency bands for rolling bearings in different conditions.

Three-layer WPT was performed on the vibration signals and energy features were extracted, as shown in Figure 12. It can be seen that the fault bearings had the largest energy factor, mainly in the 6th frequency band. According to the principle of system resonance caused by bearing fault impact, the 6th frequency bands of all signals were reconstructed into time-domain signals by wavelet packet reconstruction.

**Figure 13:**
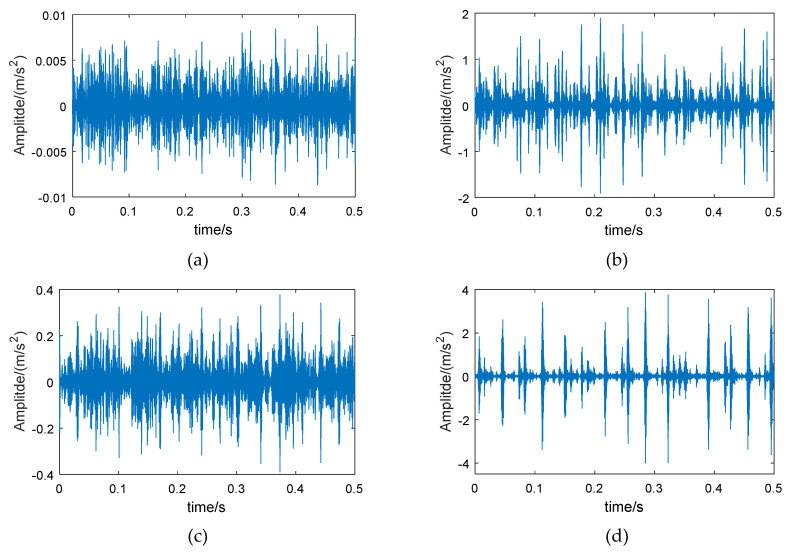
The 6th frequency band reconstructed signals of different experimental bearings (from CWRU-BDC) based on energy feature reconstruction: (**a**) normal bearing, (**b**) bearing with inner race fault, (**c**) bearing with roller fault, and (**d**) bearing with outer race fault.

**Figure 14:**
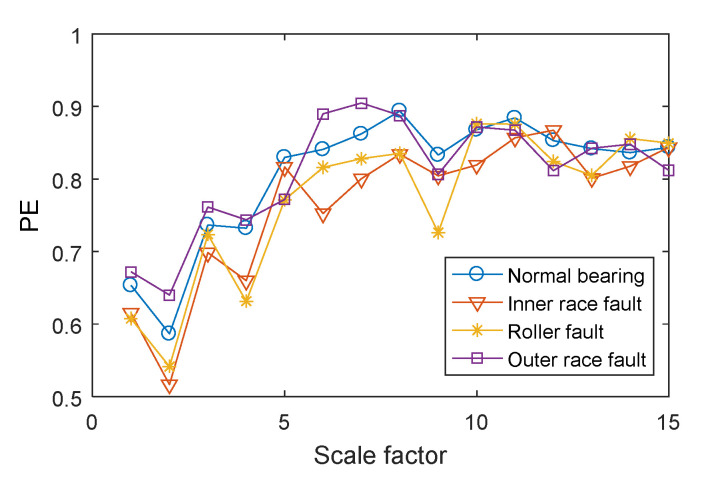
The CMPE values of rolling bearings in different conditions.

The examples of reconstructed signals are shown in Figure 13. Compared with the original signal (Figure 11), the low-amplitude noise was suppressed in fault bearing signals, and the fault impact features are more obvious. The amplitude difference between the normal bearing and the fault bearing signals was large, as a result of the vibration signal of normal bearing having a lower energy factor in the high frequency band, as well as the lower frequency band, with higher energy, having been removed. The CMPE was calculated from the reconstructed signals (Figure 14).

**Figure 15:**
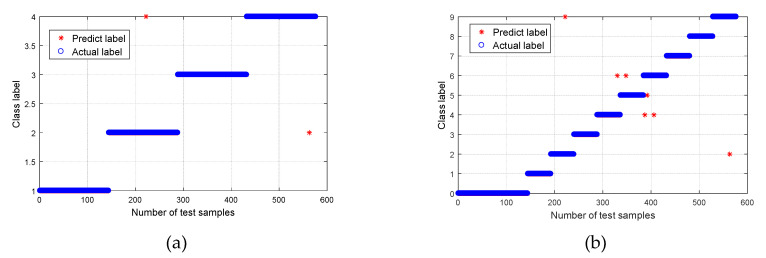
Classification results using proposed method: (**a**) Group I, with four bearing conditions; and (**b**) Group II, with 10 bearing conditions.

After obtaining the CMPE value, it was input into the MG-SVM for fault mode identification. The recognition result is shown in Figure 15. For Group I (Figure 15a), the recognition rate of normal bearings and bearings with a roller fault was 100%, the recognition rate of bearings with inner and outer race faults was 99.31%, and the average rate was 99.66%. Overall, the recognition rate was high. One of the inner race faults was incorrectly identified as an outer race fault, and one outer race fault was incorrectly identified as an inner race fault. The bearing fault point size can be further classified, and the result is shown in Figure 15b. Out of a total of 576 samples analyzed, 469 samples were correctly identified, and seven samples were recognized incorrectly. The overall recognition rate was 98.54%; however, the recognition rates of normal bearings and fault bearings were 100%. The results are listed in Table 2.

### 5.2. Performance Comparison 1

**Figure 16:**
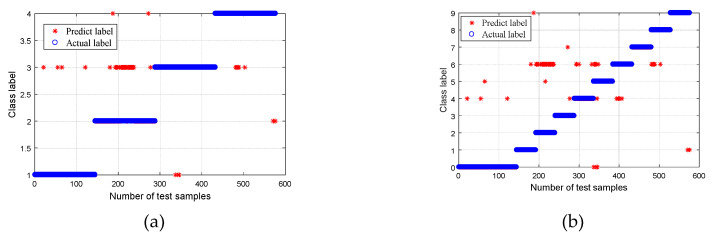
Classification results using the MPE + MG-SVM method: (**a**) Group I, with four bearing conditions; and (**b**) Group II, with 10 bearing conditions.

**Figure 17:**
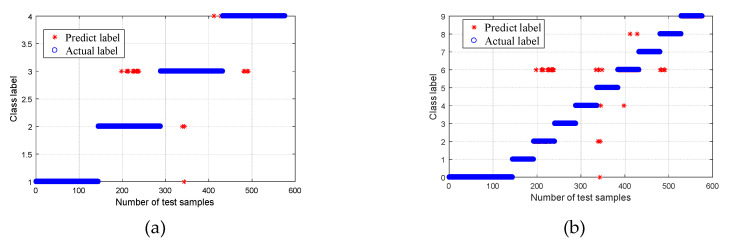
Classification results using the CMPE + MG-SVM method: (**a**) Group I, with four bearing conditions; and (**b**) Group II, with 10 bearing conditions.

**Figure 18:**
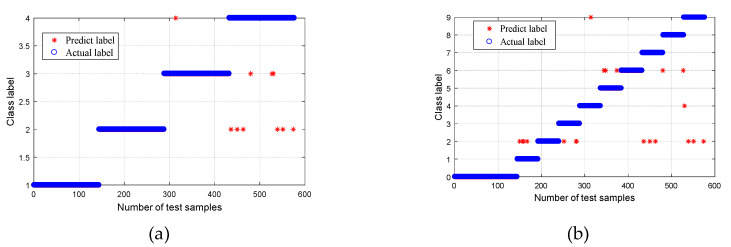
Classification results using the WPT-EF + MG-SVM method: (**a**) Group I, with four bearing conditions; and (**b**) Group II, with 10 bearing conditions.

**Figure 19:**
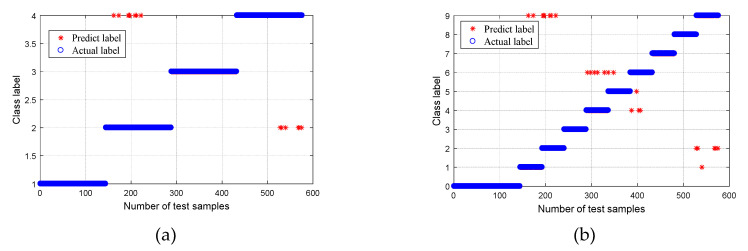
Classification results using the WPT-EFR-MPE + MG-SVM method: (**a**) Group I, with four bearing conditions; and (**b**) Group II, with 10 bearing conditions.

In order to prove the superiority of the WPT-EFR-CMPE + MG-SVM method proposed in this paper, different bearing diagnosis methods were applied to the same dataset, including MPE + MG-SVM, CMPE + MG-SVM, WPT-EF + MG-SVM, and WPT-EFR-MPE + MG-SVM, where WPT is wavelet packet transform, EFR is energy feature reconstruction, EF is energy feature, MPE is multiscale permutation entropy, CMPE is composite multiscale permutation entropy, and MG-SVM is medium Gaussian support Vector machine. The classification results are shown in Figure 16, Figure 17, Figure 18 and Figure 19.

A comparison of the fault recognition rates of the different algorithms is shown in Table 3. The proposed method was superior to the other similar diagnosis methods in both bearing fault mode classification and fault size classification, which proves that the fault feature extraction of the proposed method is more accurate, and the fault recognition rate is higher.

### 5.3. Experimental Validation 2

**Figure 20:**
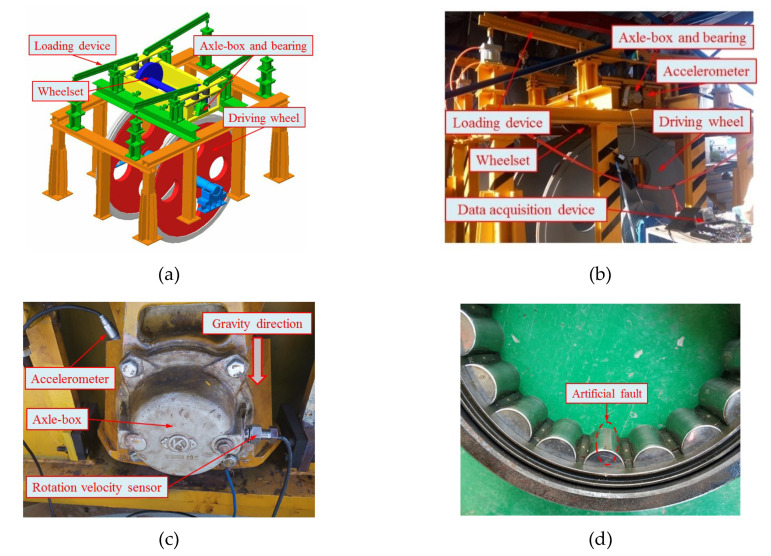
The wheelset-bearing system test bench: (**a**) schematic diagram of the test bench, (**b**) photo of overall test bench, (**c**) photo of axle-box and accelerometer, and (**d**) artificial fault on a roller.

**Figure 21:**
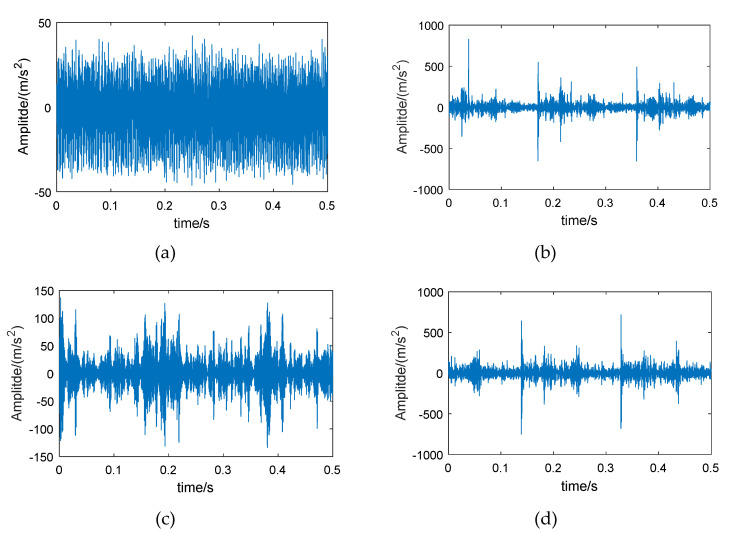
Vibration signals of different bearings (from the wheelset-bearing system test bench): (**a**) normal bearings, bearings with (**b**) inner race fault, (**c**) roller fault, and (**d**) outer race fault.

Through the wheelset-bearing system test bench, the effectiveness of the proposed method for fault diagnosis of axle-box bearing in rail vehicles was verified. The test bench is shown in Figure 20. The wheelset is driven by the driving wheel and an aerodynamic force is applied to the shaft-end axle-box through the lever, which can simulate different loads. An accelerometer was mounted in the bearing loading area of the axle-box to monitor the bearing vibration, and the monitoring direction of vibration was 45° relative to gravity direction, as shown in Figure 20c. Rotation tests of bearings in different conditions were carried out, including normal, inner race fault, roller fault, and outer race fault bearings. An artificial fault on the surface of a single roller is shown in Figure 20d. The parameters of the tested rolling bearings included rotation velocity and load, as shown in Table 4. The vibration signals were collected at a sampling frequency of 12 kHz. Figure 21 shows an example of the time-domain vibration signals for bearings in different conditions. 

**Figure 22:**
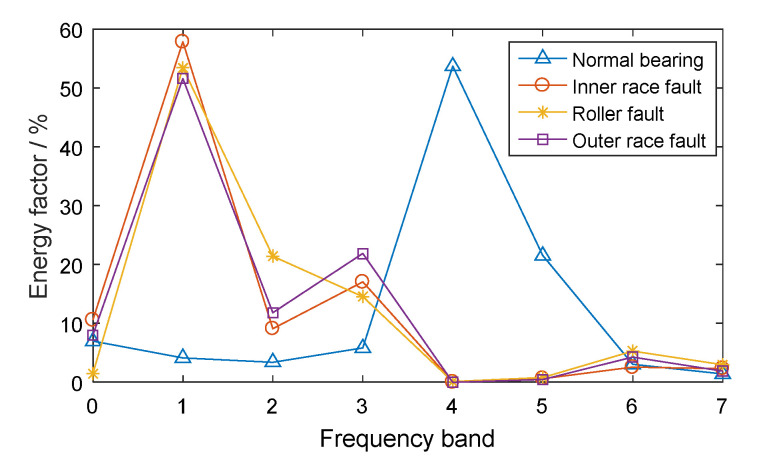
The energy factors of frequency bands of axle-box bearings in different conditions, which were calculated by three-layer WPT and energy feature extraction.

WPT was performed on the vibration signals of different condition bearings and the energy factor of each frequency band was extracted. As shown in Figure 22, the vibration energy of the fault bearing signals was mainly concentrated in the 1st frequency band, and the normal bearing signal energy was mainly concentrated in the 4th frequency band. This is because, when the rail vehicle is in operation, the wheel-rail contact generates a high-frequency vibration impact, which directly acts on the axle-box at the shaft end of the wheelset. If the axle-box bearing is a normal bearing, the accelerometer can directly monitor the high frequency impact, and if the bearing has a fault, the frequency-domain characteristics of the signal change due to the fault impact and, so, the energy distribution in the frequency domain will change as well. The 1st frequency band was selected for wavelet packet reconstruction, and the CMPE was calculated from the reconstructed time-domain signal and input into the MG-SVM classifier for bearing condition recognition.

**Figure 23:**
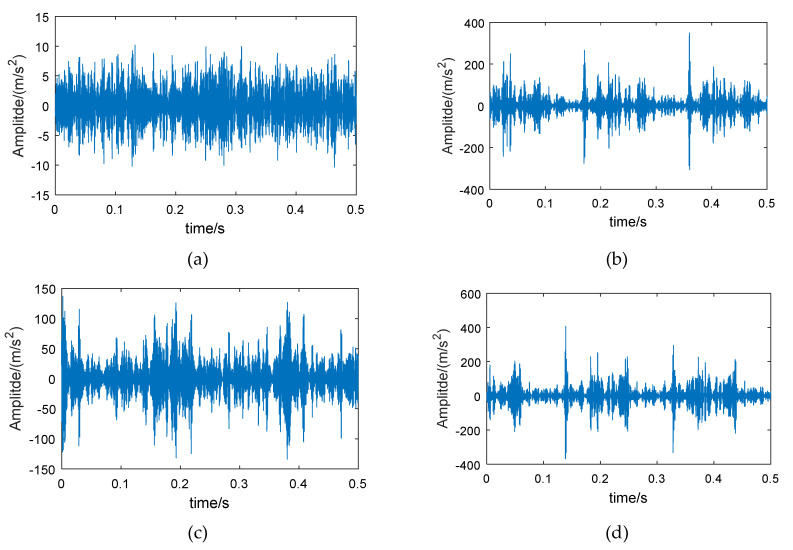
The 1st frequency band reconstructed signals of different experimental bearing (from the wheelset-bearing system test bench) based on energy feature reconstruction: (**a**) normal bearing, (**b**) bearing with inner race fault, (**c**) bearing with roller fault, and (**d**) bearing with outer race fault.

Examples of the reconstructed signals are shown in Figure 23. Compared with the original signal (Figure 21), the same conclusion can be obtained: the low-amplitude noise was suppressed in fault bearing signals, and the amplitude difference between the normal bearing and the fault bearing signal was large, which facilitates the classification of normal bearings and fault bearings. 

**Figure 24:**
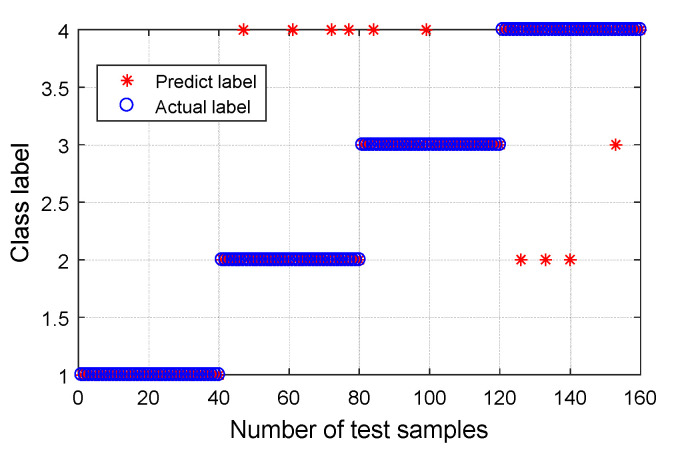
Classification results using proposed method for four bearing conditions.

For signals of different bearing condition types, the training and testing data set samples were collated. For each bearing fault type, the number of training samples was 100 and the number of testing samples was 40. For mode classification, the normal bearing was marked with label 1, the inner race fault was marked with label 2, the roller fault was marked with label 3, and the outer race fault was marked with label 4. The classification results are shown in Figure 24 and Table 5. The classification accuracy of the normal bearings and fault bearings were 100%, and the recognition rate was high. There were four inner race faults incorrectly identified as outer race faults, two roller faults incorrectly identified as outer race faults, three outer race faults incorrectly identified as inner race faults, and one outer race fault incorrectly identified as a roller fault. The overall recognition rate was 93.75%.

### 5.4. Performance Comparison 2

Based on the data set obtained from the wheelset-bearing system test bench, the performance of fault recognition between the proposed method with other bearing diagnosis methods was compared. The fault recognition rates of the different algorithms are shown in Table 6, and it can be seen that the proposed method was superior to other similar diagnosis methods in bearing fault recognition.

## 6. Discussion

Compared with the classification results of the bearing data set from CWRU-BDC, the recognition rate of the bearing data set from the wheelset-bearing system test bench was relatively low, which is due to the fact that the axle-box vibration signal collected from the wheelset-bearing system contains high-frequency interference from the wheel-rail impact and, as the wheelset hunting occurs during the traveling process, the wheel-rail impact signal transmitted to the axle-box becomes non-linear, thereby increasing the difficulty of noise separation and fault-impact feature extraction in the axle-box vibration signal, which is one of the main difficulties in fault diagnosis for axle-box bearings in rail vehicles. Considering that the bearing-localized defects cause the axle-box bearing system to resonate at a high frequency, which will lead to uneven energy distribution of the signal in the frequency domain, the frequency bands with higher energy factors contain more fault feature information. Based on the method of frequency-domain energy feature reconstruction, most of the interference signals in the original signal can be removed; in particular, the energy of the normal bearing signal was significantly reduced, which is beneficial in increasing the signal difference between a normal bearing and a fault bearing. CMPE has the characteristics of sensitivity to the impact signal and stability of the extraction results. Based on CMPE, the features of the reconstructed signals could be fully extracted. The experimental results support theoretical analysis: for different bearing test data sets, the proposed method can achieve higher recognition rate of bearing condition than the other methods. However, for higher accuracy classification of fault type and fault size in axle-box bearings of rail vehicles, further research is needed.

## 7. Conclusions

In this paper, a new method for fault diagnosis for rail vehicle axle-box bearings based on frequency-domain energy feature reconstruction, CMPE, and MG-SVM is proposed. WPT can decompose non-linear and non-stationary signals into different frequency bands. Based on the fact that fault impact can cause axle-box bearing system resonance and lead to an uneven energy distribution of the vibration signal in the frequency domain, the frequency band with maximum energy factor containing abundant fault information is reconstructed to a time-domain signal. Compared with the original signal, the reconstructed signal has lower noise interference and can better reflect the basic features of the fault signal. CMPE is better than MPE, in terms of stability, and can be used to calculate the complexity of the reconstructed signals. Multiple experimental data set analysis and classification result comparisons of different feature extraction algorithms were performed. The proposed method all achieved 100% recognition rate between normal bearings and fault bearings, and on the other hand, compared with the other methods, the proposed method can achieve higher recognition in different bearing conditions, which are 99.6% in the data set of CWRU-BDC and 93.75% in the data set of the wheelset-bearing system test bench. It was concluded that the proposed method can extract the fault feature and classify the fault mode of the bearing effectively, and this paper provides a new solution for fault diagnosis for the axle-box bearing of rail vehicles.

## Figures and Tables

**Table 1:** The classification details of the bearing experimental data set.

Bearing Condition	Fault Diameter (mm)	Number of Training Samples	Number of Testing Samples	Group II Class Label	Group I Class Label
Normal	0	36	144	0	1
IRF1	0.1778	12	48	1	2
IRF2	0.3556	12	48	2	
IRF3	0.5334	12	48	3	
BF1	0.1778	12	48	4	3
BF2	0.3556	12	48	5	
BF3	0.5334	12	48	6	
ORF1	0.1778	12	48	7	4
ORF2	0.3556	12	48	8	
ORF3	0.5334	12	48	9	

**Table 2:** The classification results of the proposed method.

Item	Fault Diameter (mm)	Accuracy (%) (Correct Number/Testing Number)				Total Accuracy (%)
		Normal	IRF	BF	ORF	
Group I	0.1778	100 (144/144)	100 (48/48)	97.9 (47/48)	100 (48/48)	98.54
	0.3556		97.9 (47/48)	97.9 (47/48)	100 (48/48)	
	0.5334		100 (48/48)	93.8 (45/48)	97.9 (47/48)	
Group II	Different diameter	100 (144/144)	99.31 (143/144)	100 (144/144)	99.31 (143/144)	99.66

**Table 3:** Comparison results of different methods for fault diagnosis accuracy (data set coming from CWRU-BDC).

Different Approaches	Accuracy (%)	
	Group I	Group II
MPE + MG-SVM	86.60	91.15
CMPE + MG-SVM	93.33	95.49
WPT-EF + MG-SVM	95.63	98.26
WPT-EFR-MPE + MG-SVM	94.79	97.57
Proposed method	98.54	99.66

**Table 4:** The parameters of the tested rolling bearing in the wheelset-bearing system test bench.

Pitch Diameter (mm)	Roller Diameter (mm)	Roller Number	Contact Angle (rad)	Rotation Velocity (rpm)	Load (kN)
176	26	18	0	300	70

**Table 5:** Classification results based on the proposed method for four bearing conditions.

Accuracy (%) (Correct Number/Testing Number)				Total Accuracy (%)
Normal	IRF	BF	ORF	
100 (40/40)	90 (36/40)	95 (38/40)	90 (36/40)	93.75

**Table 6:** Comparison results of different methods for fault diagnosis accuracy (data set from the wheelset-bearing system test bench).

Different Approaches	Accuracy (%) (Correct Number/Testing Number)				Total Accuracy (%)
	Normal	IRF	BF	ORF	
MPE + MG-SVM	100 (40/40)	65 (26/40)	88 (35/40)	83 (33/40)	83.75
CMPE + MG-SVM	100 (40/40)	73(29/40)	95 (38/40)	97 (39/40)	91.25
WPT-EF + MG-SVM	100 (40/40)	78 (31/40)	95 (38/40)	85 (34/40)	89.37
WPT-EFR-MPE + MG-SVM	95 (38/40)	63 (25/40)	90 (36/40)	83 (33/40)	82.50
Proposed method	100 (40/40)	90 (36/40)	95 (38/40)	90 (36/40)	93.75

## Appendix A

The signification of each acronym for different algorithms in this paper is listed in Table A1.

**Table A1:** The signification of each acronym for different algorithms.

Acronym	Signification
WT	Wavelet Transform
WPT	Wavelet Packet Transform
EF	Energy Feature
EFR	Energy Feature Reconstruction
PE	Permutation Entropy
MPE	Multiscale Permutation Entropy
CMPE	Composite Multiscale Permutation Entropy
EMD	Empirical Mode Decomposition
EEMD	Ensemble Empirical Mode Decomposition
CEEMD	Complementary Ensemble Empirical Mode Decomposition
CEEMDAN	Complete Ensemble Empirical Mode Decomposition with Adaptive Noise
LMD	Local Mean Decomposition
EWT	Empirical Wavelet Transform
VMD	Variational Mode Decomposition
ApEn	Approximate Entropy
SampEn	Sample Entropy
MSE	Multiscale Entropy
